# CT-Based Radiomics Signature: A Potential Biomarker for Predicting Postoperative Recurrence Risk in Stage II Colorectal Cancer

**DOI:** 10.3389/fonc.2021.644933

**Published:** 2021-03-19

**Authors:** Shuxuan Fan, Xiaonan Cui, Chunli Liu, Xubin Li, Lei Zheng, Qian Song, Jin Qi, Wenjuan Ma, Zhaoxiang Ye

**Affiliations:** ^1^Department of Radiology, Tianjin Medical University Cancer Institute and Hospital, National Clinical Research Centre of Cancer, Key Laboratory of Cancer Prevention and Therapy, Tianjin, China; ^2^School of Electronics and Information Engineering, TianGong University, Tianjin, China; ^3^Department of Colorectal Oncology, Tianjin Medical University Cancer Institute and Hospital, National Clinical Research Centre of Cancer, Key Laboratory of Cancer Prevention and Therapy, Tianjin, China; ^4^Department of Cancer Physiology, H. Lee Moffitt Cancer Center, Tampa, FL, United States; ^5^Department of Breast Imaging, Tianjin Medical University Cancer Institute and Hospital, National Clinical Research Centre of Cancer, Key Laboratory of Cancer Prevention and Therapy, Tianjin, China

**Keywords:** stage II colorectal cancer, radiomics, computed tomography, nomograms, prognosis

## Abstract

**Objective:** To evaluate whether a radiomics signature could improve stratification of postoperative risk and prediction of chemotherapy benefit in stage II colorectal cancer (CRC) patients.

**Material and Methods:** This retrospective study enrolled 299 stage II CRC patients from January 2010 to December 2015. Based on preoperative portal venous-phase CT scans, radiomics features were generated and selected to build a radiomics score (Rad-score) using the Least Absolute Shrinkage and Selection Operator (LASSO) method. The minority group was balanced by the synthetic minority over-sampling technique (SMOTE). Predictive models were built with the Rad-score and clinicopathological factors, and the area under the curve (AUC) was used to evaluate their performance. A nomogram was also constructed for predicting 3-year disease-free survival (DFS). The performance of the nomogram was assessed with a concordance index (C-index) and calibration plots.

**Results:** Overall, 114 features were selected to construct the Rad-score, which was significantly associated with the 3-year DFS. Multivariate analysis demonstrated that the Rad-score, CA724 level, mismatch repair status, and perineural invasion were independent predictors of recurrence. Results showed that the Rad-score can classify patients into high-risk and low-risk groups in the training cohort (AUC 0.886) and the validation cohort (AUC 0.874). On this basis, a nomogram that integrated the Rad-score and clinical variables demonstrated superior performance (AUC 0.954, 0.906) than the clinical model alone (AUC 0.765, 0.705) in the training and validation cohorts, respectively. The C-index of the nomogram was 0.872, and the performance was acceptable.

**Conclusion:** Our radiomics-based model can reliably predict recurrence risk in stage II CRC patients and potentially provide complementary prognostic value to the traditional clinicopathological risk factors for better identification of patients who are most likely to benefit from adjuvant therapy. The proposed nomogram promises to be an effective tool for personalized postoperative surveillance for stage II CRC patients.

## Introduction

Colorectal cancer (CRC) is the fourth leading cause of cancer-related death and approximately one-quarter of CRC patients are diagnosed as stage II (1). Surgical resection is recommended as the first choice for the treatment in stage II CRC (2). Nevertheless, 20–25% of these patients show fatal disease recurrence after surgery (3). Current clinical guidelines (4, 5) recommend adjuvant chemotherapy for stage II CRC patients with any poor prognostic features, including T4 lesions, poorly differentiated tumors, obstruction or perforation, lymphovascular invasion (LVI), perineural invasion (PNI), positive margins, and a small number (<12) of lymph nodes examined after surgery. However, the accuracy of these clinicopathological risk factors is unsatisfactory to identify high risk recurrence patients and determine the indication for adjuvant chemotherapy (6). Furthermore, due to the considerable short- and long-term toxicities (7, 8), the benefit of adjuvant chemotherapy remains controversial, and not all patients derive clinical benefit (9–11). Therefore, approaches that can accurately predict the recurrence risk of CRC preoperatively to tailor patient treatment and improve long-term patient survival are urgently needed.

Recently, several poenttial molecular and immune predictors of recurrence risk have been investigated, such as microRNA, FOXP3+ tumor-infiltrating lymphocytes (TILs) (12, 13), but these biomarkers still require further validation and are not used routinely in clinical practice. Moreover, the National Comprehensive Cancer Network (NCCN) (2) asserts that there are insufficient data to recommend the use of multigene assays to predict the prognosis of CRC at early stage.

Radiomics, as an emerging quantitative technique, has shown great potential to characterize intra-tumor heterogeneity and improve prognosis prediction in various types of cancer (14–20). Based on the successful application of radiomics analyses in the area of precision oncology, we hypothesized that a novel radiomics methodology could provide an accessible method for patient selection, leading to improved outcomes.

Accordingly, this study aims to develop and validate a CT-based radiomics analysis model to predict postoperative recurrence risk in stage II CRC patients. Moreover, we developed a novel nomogram in conjunction with this radiomics signature and revealed common clinicopathological risk factors associated with disease recurrence to help better determine adjunctive treatment approaches, and providing individualized treatment as well as evaluation follow-up planning for CRC patients in stage II.

## Methods and Materials

### Patients

**Figure 1:**
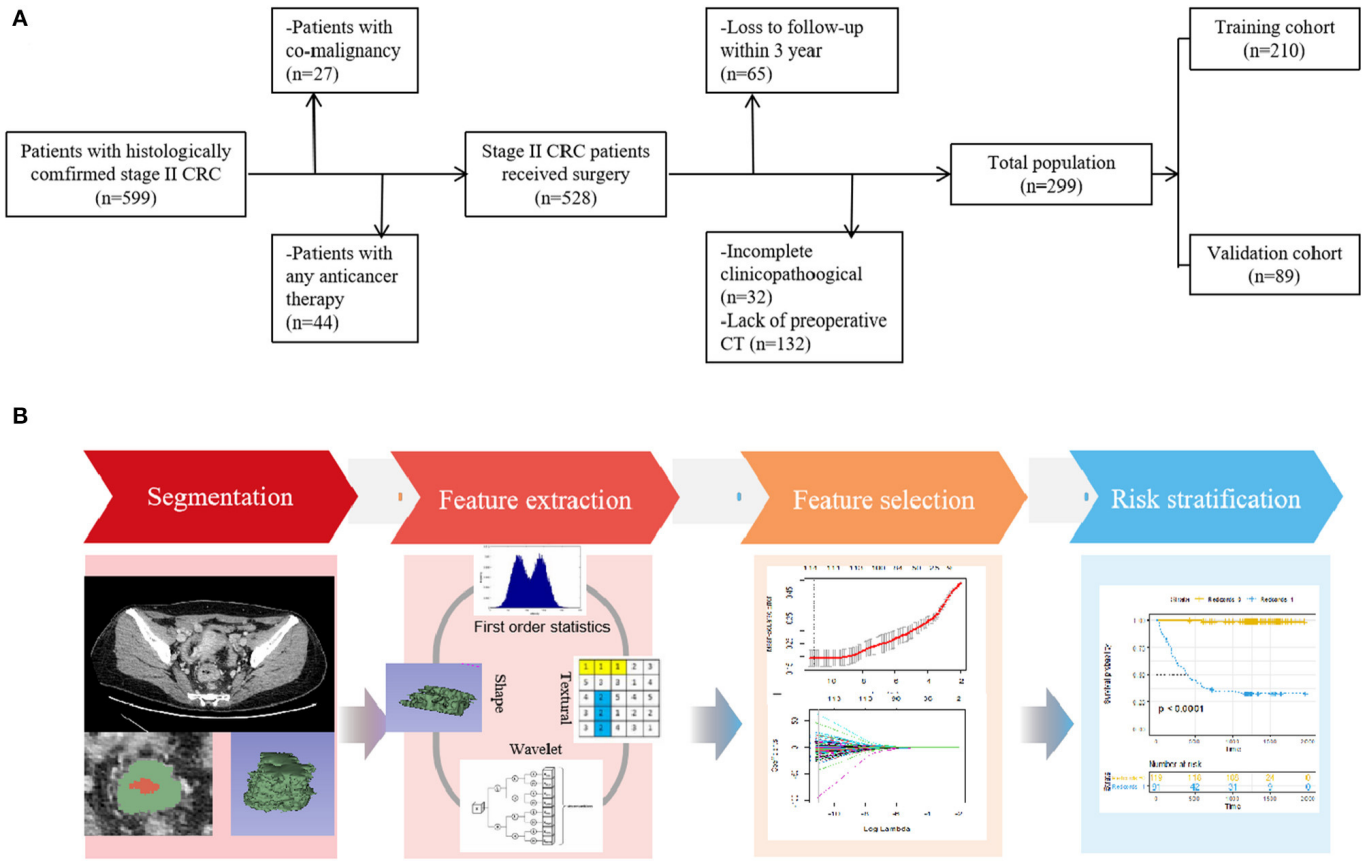
**(A)** Flowchart of the patient selection process in the present study. **(B)** Study workflow. The workflow illustrated an overview of the tumor segmentation, feature extraction process, and analysis.

With approval from the local ethics committee of our hospital, this retrospective study waived the requirement for informed consent. The study design is illustrated in Figure 1.

From January 2010 to December 2015, consecutive patients with histologically proven stage II CRC and available follow-up information were included. All patients received surgucal resection within 2 weeks after preoperative CT scan. The exclusion criteria were as follows: (1) patients previously treated with any anticancer therapy (*n* = 44), (2) patients with co-malignancy (*n* = 27), (3) patients with lack of available baseline demographics and CT images (*n* = 132), and (4) patients lost to follow-up within 3 years (*n* = 65) and those with incomplete clinicopathological data (*n* = 32).

Consequently, 299 patients (median [interquartile range {IQR}] age, 59 [30–82] years) were enrolled in our study, including 187 male (median [IQR] age, 59 [31–82] years) and 112 female (median [IQR] age, 58 [30–80]; Table 1). For temporally independent validation, patients were randomly separated into two cohorts at a 7 to 3 ratio: 210 for training and 89 for validation.

**Table 1:** Baseline clinical and pathologic characteristics of patients.

**Characteristics**	**All patients (*N* = 299)**	**Training corhort (*N =* 210)**	**Validation corhort (*N =* 89)**
**Gender**, ***n*** **(%)**			
Male	187 (62.5%)	133 (63.3%)	54 (60.7%)
Female	112 (37.5%)	77 (36.7%)	35 (39.3%)
**Age**, ***n*** **(%)**			
>65	88 (29.4%)	62 (29.5%)	26 (29.2%)
≤ 65	211 (70.6%)	148 (70.5%)	63 (70.8%)
**Histologic grade**, ***n*** **(%)**			
Well	43 (14.4%)	32 (15.2%)	11 (12.4%)
Moderate	165 (55.2%)	116 (55.2%)	49 (55.1%)
Poor	91 (30.4%)	62 (29.6%)	29 (32.5%)
**Location**, ***n*** **(%)**			
Ascending colon	43 (14.4%)	31 (14.8%)	12 (13.5%)
Transverse colon	7 (2.3%)	4 (1.9%)	3 (3.4%)
Descending colon	11 (3.7%)	8 (3.8%)	3 (3.4%)
Sigmoid colon	56 (18.7%)	42 (20.0%)	14 (15.7%)
Rectum	182 (60.9%)	125 (59.5%)	57 (64.0%)
**Smoking**, ***n*** **(%)**			
No	244 (81.6%)	174 (82.9%)	70 (78.7%)
Yes	55 (18.4%)	36 (17.1%)	19 (21.3%)
**Hypertension**, ***n*** **(%)**			
No	223 (74.6%)	154 (73.3%)	69 (77.5%)
Yes	76 (25.4%)	56 (26.7%)	20 (22.5%)
**Family history of cancer**, ***n*** **(%)**			
No	254 (84.9%)	180 (85.7%)	74 (83.1%)
Yes	45 (15.1%)	30 (14.3%)	15 (16.9%)
**Diabetes**, ***n*** **(%)**			
No	277 (92.6%)	201 (95.7%)	76 (85.4%)
Yes	22 (7.4%)	9 (4.3%)	13 (14.6%)
**CEA level**, ***n*** **(%)**			
Normal	172 (57.5%)	121 (57.6%)	51 (57.3%)
Abnormal	127 (42.5%)	89 (42.4%)	38 (42.7%)
**CA242**, ***n*** **(%)**			
Normal	229 (76.6%)	160 (76.2%)	69 (77.5%)
Abnormal	70 (23.4%)	50 (23.8%)	20 (22.5%)
**CA724**, ***n*** **(%)**			
Normal	230 (76.9%)	159 (75.7%)	71 (79.8%)
Abnormal	69 (23.1%)	51 (24.3%)	18 (20.2%)
**CA199**, ***n*** **(%)**			
Normal	237 (79.3%)	166 (79.0%)	71 (79.8%)
Abnormal	62 (20.7%)	44 21.0%)	18 (20.2%)
**Ki-67 level**, ***n*** **(%)**			
Low	13 4.3%)	10 (4.8%)	3 (3.4%)
High	286 (95.7%)	200 (95.2%)	86 (96.6%)
**T stage**, ***n*** **(%)**			
T3	188 (62.9%)	132 (62.9%)	56 (62.9%)
T4	111 (37.1%)	78 (37.1%)	33 (37.1%)
**Lymphovascular invasion**, ***n*** **(%)**			
Absent	241 (80.6%)	171 (81.4%)	70 (78.7%)
Present	58 (19.4%)	39 (18.6%)	19 (21.3%)
**Perineural invasion**, ***n*** **(%)**			
Absent	271 (90.6%)	187 (89.0%)	84 (94.4%)
Present	28 (9.4%)	23 (11.0%)	5 (5.6%)
**IOP status**, ***n*** **(%)**			
No	272 (91.0%)	191 (91.0%)	81 (91.0%)
Yes	27 (9.0%)	19 (9.0%)	8 (9.0%)
**Number of nodes examined**, ***n*** **(%)**			
12 or more	258 (86.3%)	181 (86.2%)	77 (86.5%)
<12	41 (13.7%)	29 (13.8%)	12 (13.5%)
**Mismatch repair status**, ***n*** **(%)**			
MSI-L	240 (80.3%)	172 (81.9%)	68 (76.4%)
MSI-H	59 19.7%)	38 (18.1%)	21 (23.6%)
**Adjuvant chemotherapy**, ***n*** **(%)**			
No	114 (38.1%)	85 (40.5%)	29 (32.6%)
Yes	185 (61.9%)	125 (59.5%)	60 (67.4%)

*MSI, microsatellite instability; CEA, carcinoembryonic antigen; CA242, carbohydrate antigen 242; CA724, carbohydrate antigen 724; CA199, carbohydrate antigen199; IOP status, internal obstruction or perforation*.

### Risk Factors

In this study, the clinicopathological risk factors included gender, age, T stage, CT-reported tumor location (Ascending colon, Transverse colon, Descending colon, Sigmoid colon, and Rectum), histologic grade of the tumor, smoking history, hypertension history, diabetes history, family history of cancer, internal obstruction, or perforation (IOP) status, number of lymph nodes examined (≥12 vs. <12), LVI status, PNI status, mismatch repair status, Ki-67 expression level, and history of postoperative adjunctive chemotherapy (present/absent). Laboratory analysis included tests for carcinoembryonic antigen (CEA) with the values of 5 ng/mL, carbohydrate antigen 724 (CA724) with 6 U/mL, carbohydrate antigen 242 (CA242) with 20 U/mL and carbohydrate antigen 199 (CA199) with 39 U/mL. The histologic grade was on the basis of the World Health Organization (WHO) classification of the digestive system tumors, 4th edition (21). Ki-67 was identified as positive expression when staining was equal to or above 40% of the specimen, while <40% denoted negativity (22). DNA mismatch repair (MMR) status was assessed by immunohistochemical staining for MMR gene protein products (MLH1, MSH2, MSH6, and PMS2) expression (23). The loss of an MMR protein in tumors was defined as high-frequency microsatellite instability (MSI-H), whereas intact MMR proteins in tumors was defined as low-frequency MSI (MSI-L). These datasets were obtained from the institutional archives.

### Follow-Up

After surgical resection, all patients were followed-up for at least 3 years. Patients were followed-up by recording their CEA levels and evaluating contrast CT images in the first month after surgery and every 3–6 months thereafter. The end-point was time to recurrence, which was defined as the prognostic performance of the imaging features for distant metastasis, local recurrence or an atypical finding with histopathological confirmation.

DFS was calculated from the date of surgery until either the date of confirmed clinical recurrence or time of last available contact.

### CT Scan Acquisition and Tumor Segmentation

The pretreatment abdominal with/without pelvic contrast-enhanced CT scans were obtained using a varied set of CT scanners (Supplementary Material 1).

For tumor segmentation, all portal venous-phase CT images with DICOM format were retrieved from the picture archiving and communication system (PACS) at our institution, because of well-differentiation between tumor tissue and adjacent normal bowel wall. Volume of interests (VOIs) were semiautomatically delineated using the open-source software 3D Slicer (version 4.9, www.slicer.org). This was performed by a board-certified radiologist (reader 1, C.X.N.) with 6 years of experience in abdominal radiology and then reviewed and modified by another experienced radiologist (reader 2, F.S.X., with 10 years of experience in abdominal diagnosis); both were blinded to the clinicopathologic and outcome details. To assess feature reproducibility, segmentation was repeated in 20 randomly selected patients by another radiologist (reader 3, S.Q., with 10 years of experience in abdominal diagnosis). On the basis of the feature extraction by reader 1 and reader 3, the interobserver ICCs were calculated. Additionally, reader 1 repeated the assessment of the same 20 randomly selected patients 1 month later to evaluate intraobserver reproducibility.

### Feature Selection and Model Construction

After tumor segmentation, 1561 radiomics features (RFs) per case were extracted from the region of interest (ROI) with the open-source python package “Pyradiomics V1.3.0” (http://www.radiomics.io/pyradiomics.html). The following radiomic features were included for feature computation: (1) Shape-based features; (2) First-order statistics features; (3) gray level co-occurrence matrix-based features (GLCM); (4) gray level run-length matrix-based features (GLRLM); (5) gray level size zone matrix (GLSZM); (6) neighboring gray tone difference matrix (NGTDM), and (7) gray level dependence matrix (GLDM). The detailed feature extraction methodology was shown in Supplementary Material 2.

A two-step procedure of feature selection was performed for dimensionality reduction. Firstly, we calculated the intra- and interclass correlation coefficients (ICCs) for all 1561 radiomics features based on the abovementioned resegmentations to remove the unstable features. Only features with both intra- and interobserver ICC values >0.90 were initially selected. Secondly, considering that the extracted features were high dimensional, three steps were adopted to reduce the dimension of the features: (1) Kruskal-Wallis test was first used to screen the image features with statistically significant differences (*P* < 0.05). The objective is to remove the irrelevant or poorly correlated characteristic parameters. (2) In consideration of the possibility of repeated expression of lesion information among features, Spearman analysis was adopted as the correlation analysis to remove image features with correlations <0.8. (3) The Least Absolute Shrinkage and Selection Operator (LASSO) logistic regression algorithm (24, 25), with penalty parameter tuning conducted by 10-fold cross validation, was used to preidentify the top-ranked features. The candidate predictive features with a zero-fit weight were selected. Thereafter, a Rad-score for each outcome was built via a linear combination of selected features and coefficient vectors. The Rad-score cutoff values for the classification of high- and low-risk groups were chosen according to the Youden index criterion (26).

All radiomics feature selection and model construction processes were performed in the training cohort and then evaluated in the validation cohort.

### Statistical Analysis

Chi-square test, Student's *t*-test, Fisher's exact test and Mann-Whitney *U*-test were used for categorical and continuous variables, where appropriate. For survival analyses, we used the Kaplan-Meier method to analyze DFS and the log-rank test to compare survival curves. A multivariate logistic regression analysis with Cox regression coefficients was performed to construct nomograms combining clinicopathological factors and RFs. To evaluate the predictive accuracy of the nomograms, a calibration curve with bootstrapping, which measured the agreement between the nomogram-predicted outcome probability and the average actual probability, was plotted. The performance of the clinical model, radiomics signature and combined model were evaluated by diagnostic accuracy, sensitivity, specificity, and the time-dependent receiver operating characteristic (ROC) curve. Differences between various AUCs were compared with the DeLong test. All statistical analyses were performed using R software (version 6.1, www.r-project.org). A two-tailed *P* < 0.05 was considered a statistically significant difference. Due to sample imbalance (the number of recurrences was much smaller than that of nonrecurrences), the synthetic minority over-sampling technique (SMOTE) (27, 28) was used to overcome classification bias in favor of the majority class. Further details are included in Supplementary Material 3.

## Results

### Patient Characteristics

The detailed clinicopathological characteristics and demographics of patients in the training and validation cohorts are shown in Table 1. The median DFS time was 1,024 days (range, 23–1,978 days), and 81 (27.1%) of 299 patients experienced recurrence during the follow-up period.

### Radiomics Feature Selection and Prognostic Radiomic Classifiers

**Figure 2:**
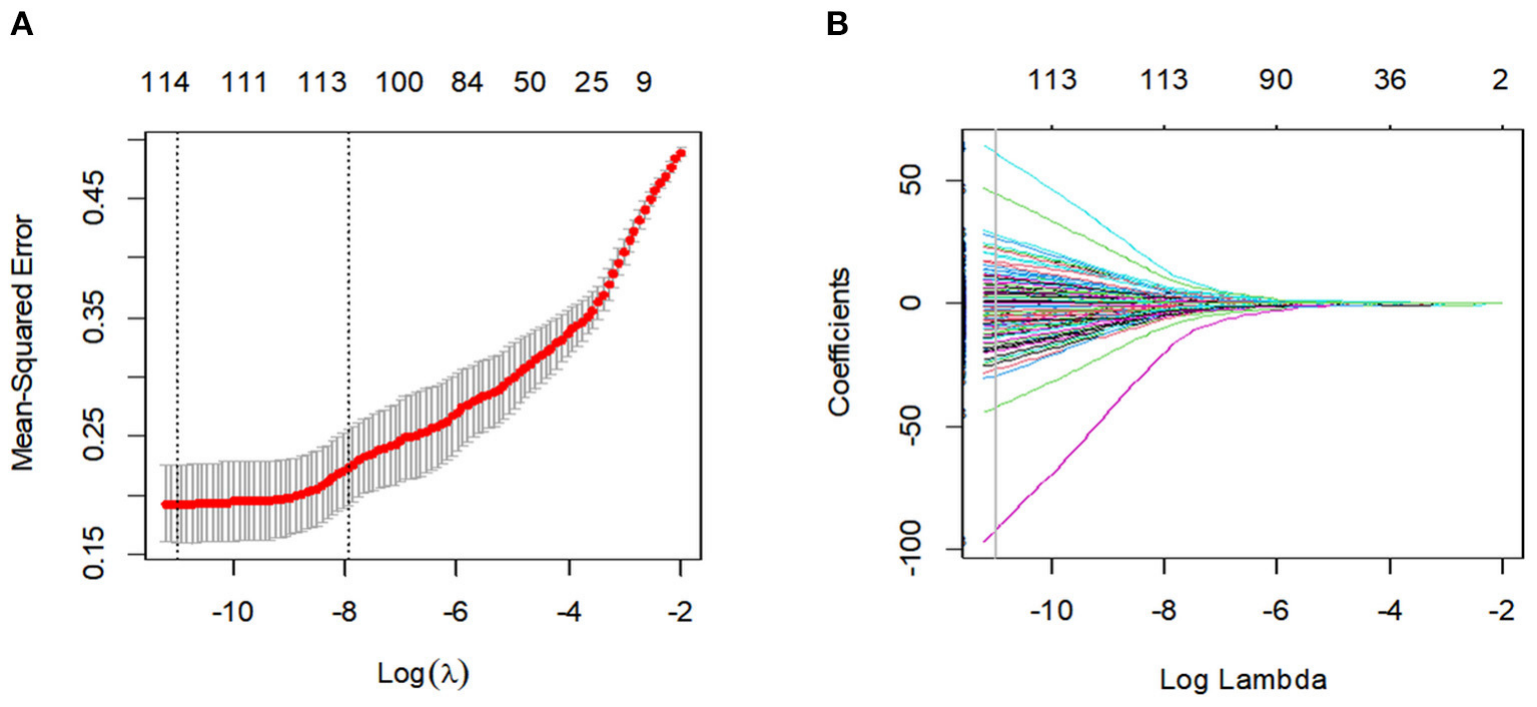
Feature selection using the least absolute shrinkage and selection operator (LASSO) binary logistic regression model. **(A)** Tuning parameter (Lambda) selection in the LASSO model used 10-fold cross-validation via minimum criteria. The gray line in the figure is the partial likelihood estimate corresponding to the optimal value of lambda. The optimal lambda value of 1.638236e-05 was chosen. **(B)** LASSO coefficient profiles of the 114 features. A vertical line was plotted at the optimal lambda value, which resulted in ten features with non-zero coefficients.

After the feature robustness analysis, we adopted the 118 most stable features with both intra- and interobserver ICC values >0.90 for further analysis. Then, the 114 most significant features selected by the LASSO logistic regression analysis were adopted in the Rad-score calculation formula (Figure 2, Supplementary Table 1). The Rad-score calculation formulas are listed in Supplementary Material 4.

**Figure 3:**
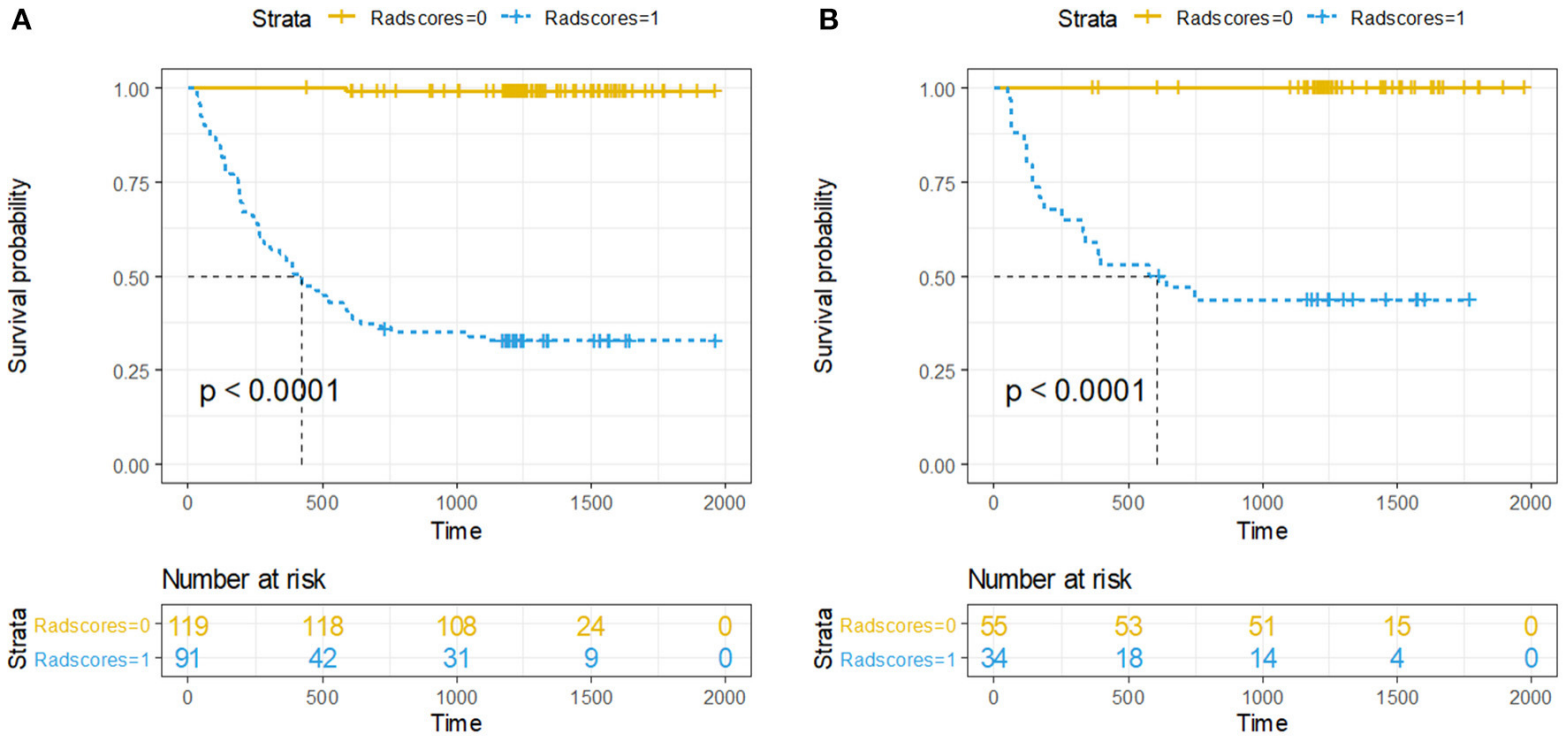
Kaplan-Meier curves of DFS between high-risk and low-risk groups stratified by the radiomics-based classifier, **(A)** Training cohort. **(B)** Validation cohort. *P*-values were calculated using the log-rank test.

Accordingly, Rad-score of 0.374 was calculated to differentiate between high-risk and low-risk group. Ninety-one (43.3%) patients were assigned to the high-risk group, and among them, 61 (67.0%) had developed recurrence by the overall endpoint. A total of 119 (56.7%) patients were assigned to the low-risk group, and among them, 1 (0.8%) had developed recurrence by the overall endpoint. The recurrence risk in the high-risk group was significantly higher than the low-risk group (odds ratio 239.933, *P* < 0.001). We performed the same analyses in the validation cohort. The 3-year DFS was 55.9% for the high-risk group and 0% for the low-risk group (HR 130, 95% CI 18–940; *P* < 0·0001; Figure 3).

### Integration With Clinical Features

Univariate Cox regression analysis in the training cohort showed that the Rad-score, CA242, CA724, CA199, and CEA levels, mismatch repair status, T stage, LVI, PNI, IOP status, number of lymph nodes examined, and adjuvant chemotherapy were significant prognostic factors for DFS (Table 2). Subsequently, all the relevant factors were entered into the multivariate Cox analysis, and the Rad-score, CA724 level, mismatch repair status, and PNI were considered independent risk factors for model building (Table 2). Detailed results of the validation cohort analyses are listed in Supplementary Table 2.

**Table 2:** Univariate and multivariate cox regression analysis of disease-free survival in the training cohort.

**Variable**	**Univariate analysis**			**Multivariate analysis**		
	**HR**	**95%CI**	***P*-value**	**HR**	**95%CI**	***P*-value**
Gender	1.181	0.71–2	0.521			
Age	1.011	0.99-1	0.378			
Histologic grade	1.155	0.79–1.7	0.465			
Location	1.001	0.84–1.2	0.993			
Smoking	1.222	0.65–2.3	0.533			
Hypertension	0.607	0.32–1.1	0.120			
Family history of cancer	1.010	0.5–2	0.978			
Diabetes	1.912	0.69–5.3	0.210			
CEA level	3.095	1.8–5.2	<0.001^*^	1.423	0.737–2.747	0.293
CA242	2.873	1.7–4.8	<0.001^*^	0.898	0.361–2.234	0.818
CA724	2.185	1.3–3.7	0.003^*^	2.069	1.055–4.057	0.034^*^
CA199	2.914	1.7–4.9	<0.001^*^	1.759	0.687–4.501	0.239
Ki−67 level	0.998	0.98–1	0.817			
T stage	1.688	1–2.8	0.039	1.372	0.701–2.685	0.356
Lymphovascular invasion	2.486	1.5–4.2	<0.001^*^	0.861	0.438–1.691	0.664
Perineural invasion	4.673	2.7–8.1	<0.001^*^	3.566	1.748–7.275	<0.001^*^
IOP status	2.763	1.5–5.2	0.0016^*^	1.777	0.835–3.781	0.135
Number of nodes examined	4.187	2.4–7.2	<0.001^*^	1.327	0.720–2.445	0.365
Mismatch repair status	0.264	0.096–0.73	0.010^*^	0.227	0.075–0.683	0.008^*^
Adjuvant chemotherapy	2.140	1.2–3.8	0.0088^*^	0.497	0.229–1.077	0.076
Rad–score	32	13–77	<0.001^*^	44.255	16.369–119.447	<0.001^*^

*CEA, carcinoembryonic antigen; CA242, carbohydrate antigen 242; CA724, carbohydrate antigen 724; CA199, carbohydrate antigen199; IOP status, internal obstruction or perforation*.

* *P < 0.05*.

**Figure 4:**
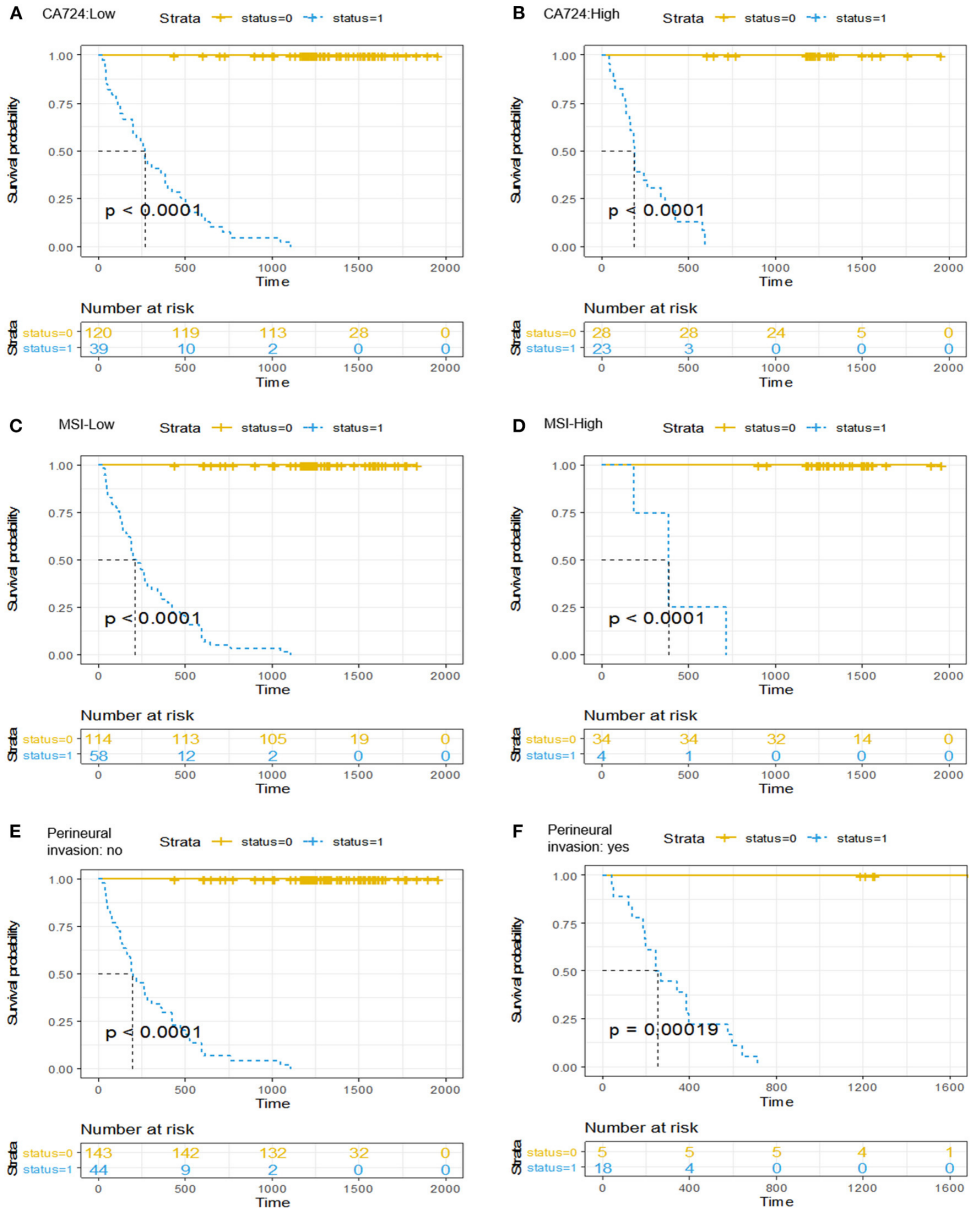
Kaplan-Meier curves of the survival analysis in patients stratified according to the radiomics-based classifier by clinicopathological risk factors. **(A,B)** CA724. **(C,D)** Mismatch repair status. **(E,F)** Perineural invasion. We calculated *P*-values using the log-rank test.

It should be emphasized that after a multivariable analysis was performed to adjust for the clinicopathological risk factors, the radiomics-based classifier remained a powerful and independent factor in both the training and validation cohorts (HR 38.08; 95% CI: 14.45–100.33, *P* < 0.001; HR 60.72; 95% CI: 6.62–557.15, *P* < 0.001). When stratified by different clinicopathological risk factors, patients in the low-risk group significantly showed better 3-year DFS than those in the high-risk group (Figure 4).

Surprisingly, the multivariable cox regression analysis showed that adjuvant chemotherapy was not an independent risk predictor in the training (HR 0.497, 95% CI 0.229–1.077; *P* = 0.076; Table 2) and validation sets (HR 2.560, 95% CI 0.429–15.289, *P* = 0.303; Supplementary Table 2). However, our radiomics-based classifier indicated that patients in the high-risk group derived a greater survival benefit from adjuvant chemotherapy (HR 0.303, 95% CI 0.181–0.506, *P* < 0.001; Supplementary Figure 1).

### Patient Risk Stratification

Table 3 presents the performance results obtained in the training and validation cohorts for the clinical, radiomics, and radiomics plus clinical models.

**Table 3:** Predictive performance for the proposed models.

**Different models**	**Training dataset** ***N=*** **210**				**Validation dataset** ***N=*** **89**			
	**AUC (95%CI)**	**ACC**	**SENS**	**SPEC**	**AUC (95%CI)**	**ACC**	**SENS**	**SPEC**
Clinical model	0.756 (0.694–0.817)	75.2%	82.0%	58.3%	0.705 (0.586–0.823)	77.5%	84.7%	47.1%
Radiomics signature	0.886 (0.840–0.931)	85.2%	99.2%	67.0%	0.874 (0.802–0.945)	84.3%	100%	57.6%
Combined model	0.954 (0.930–0.978)	88.6%	99.2%	72.6%	0.906 (0.844–0.9680)	87.6%	98.4%	64.3%

*AUC, area under the curve; CI, confidence interval; ACC, accuracy; SENS, sensitivity; SPEC, specificity*.

**Figure 5:**
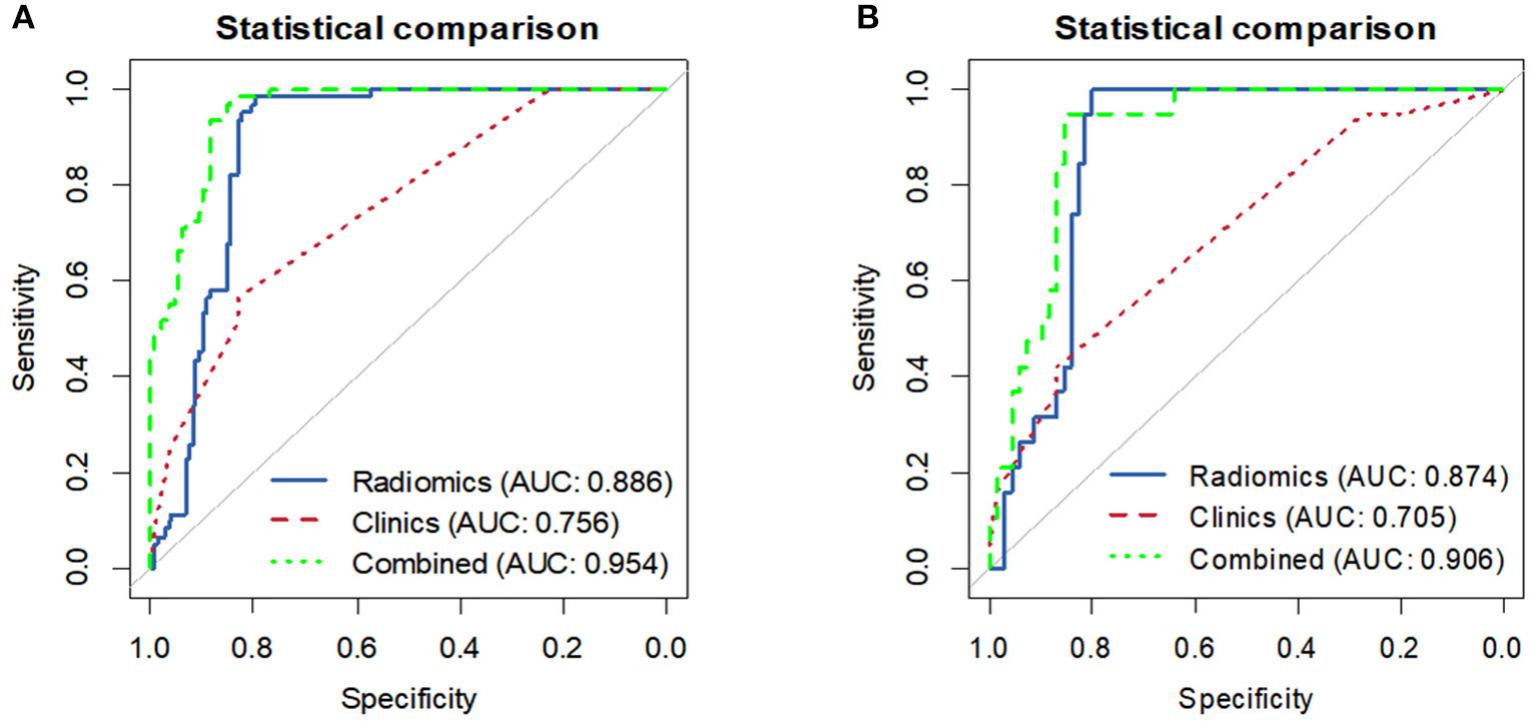
The ROC curves comparing the performance of the Clinical, Radiomics signature and combined model in predicting the recurrence risk for patients with stage II CRC. **(A)** Training cohort. **(B)** Validation cohort.

Overall, in the stratification analysis, the Rad-score showed a significant discrimination of high-risk and low-risk patients with CRC in the training cohort (AUC 0.886, 95% CI 0.840–0.931) and the validation cohort (AUC 0.874, 95% CI 0.802–0.945). The combined model yielded an AUC of 0.954 (95% CI 0.930–0.978) in the training cohort and 0.906 (95% CI 0.844–0.968) in the validation cohort, and these values were significantly greater than those obtained the model of clinical parameters alone (AUC 0.756, 95% CI 0.694–0.817, *P* < 0.001; AUC 0.704, 95% CI 0.586–0.823, *P* < 0.001) (Figure 5). Comparison of the performance demonstrated no significance difference between the training and validation cohorts (*P* = 0.160). Thus, the radiomics classifier could add prognostic value when combined with clinicopathological prognostic features.

### Development and Validation of the Nomogram

**Figure 6:**
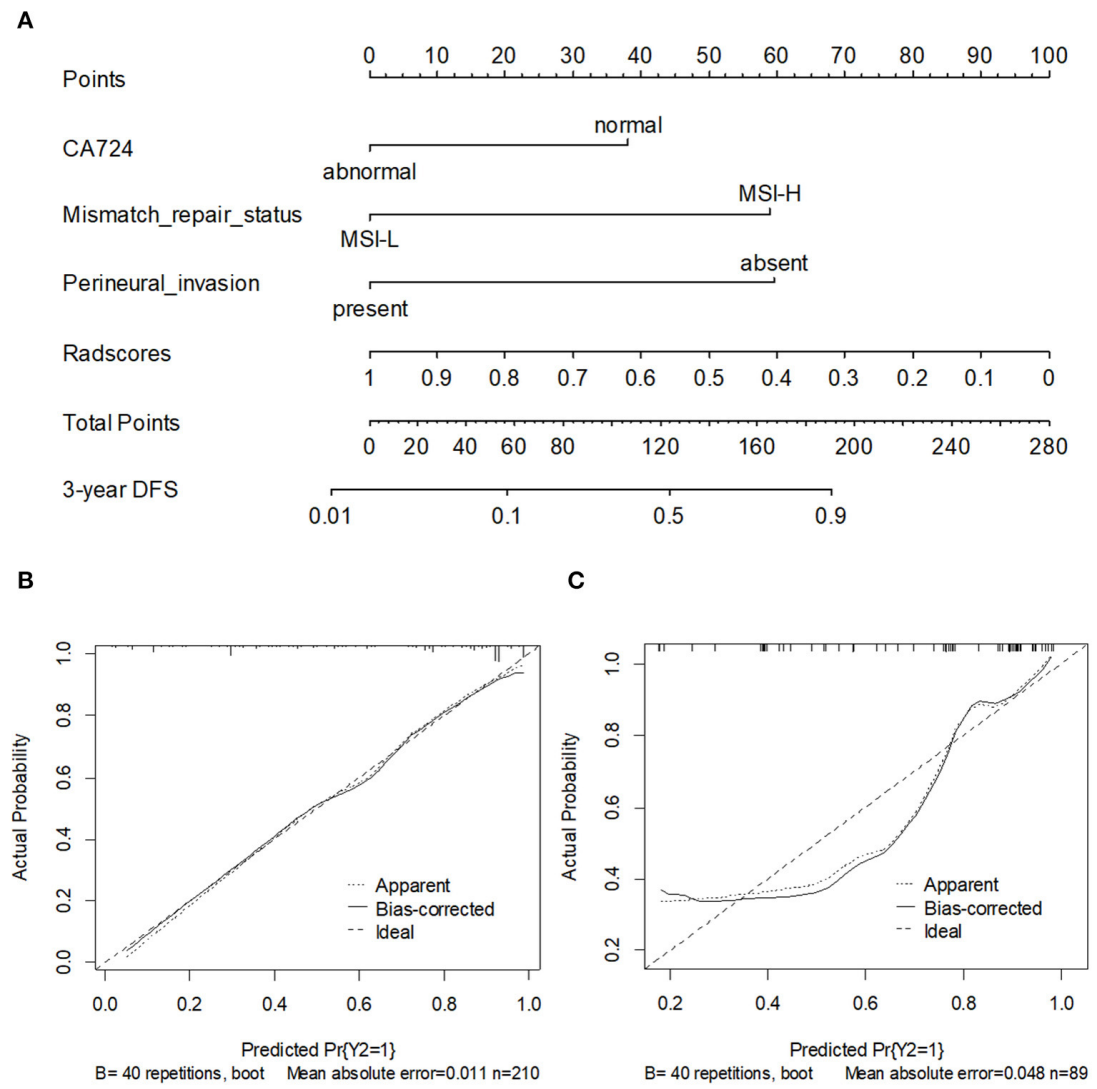
**(A)** A nomogram for 3-year DFS was developed in the training data set with clinicopathological characteristics. Calibration curves of the nomogram in training cohort **(B)** and validation cohort. **(C)** Model performance is shown by the plot, relative to the 45-degree line, which represents perfect prediction.

To provide the physicians with a simple and quantitative approach to predict the disease recurrence probabilities for each individual patient, we developed a combined nomogram that integrated both the Rad-score and clinicopathological risk factors (Figure 6A). Notably, for the Rad-score, the variable with the largest coefficient absolute value was set as a reference, whose scale ranged was from 0 to 100. The calibration curve of the combined nomogram showed good agreement between the nomogram prediction and actual observation (Figures 6B,C). The nomogram was able to accurately predict the 3-year DFS, with a C-index of 0.872.

## Discussion

In this retrospective study, a radiomics-based model was established to predict postoperative recurrence risk in stage II CRC patients. We showed that the radiomics signature is an independent risk predictor and could serve as a non-invasive biomarker for patient stratification. Furthermore, a simple-to-use nomogram incorporating the radiomics signature and clinical variables achieved significantly better performance than the clinical prediction model alone. To the best of our knowledge, this is the first study to use a CT-based radiomics biomarker to evaluate the risk of postoperative recurrence and to determine benefits derived from adjuvant chemotherapy in stage II CRC patients.

Understanding the postoperative recurrence risk in stage II CRC patients plays a central role in directing personalized therapeutic regimen selection and devising targeted surveillance follow-up protocols. Hoshino et al. (29) established a predictive nomogram of recurrence in stage II CRC, with a C-index of 0.64, based only on the following clinical characteristics: sex, tumor depth, tumor location, CEA level, LVI, and number of lymph nodes examined. However, these clinicopathological risk factors are not sufficiently accurate to identify patients at a high risk of recurrence and are not precise predictors that can be used to evaluate the intra-tumoral heterogeneity in routine practice (30, 31). Therefore, it is necessary to add prognostic value to the current staging system so that it can quantify heterogeneity within tumors for further analysis and identify patients who could derive the greatest therapeutic benefit from adjuvant chemotherapy.

Radiomics, an emerging field within medical imaging, is now regarded as a potential powerful approach that can quantify tumor heterogeneity and facilitate better clinical decision making (32–34). In the current study, we determined the Rad-score, which demonstrated a strong ability to predict the risk of recurrence in stage II CRC and performed better prediction performance than the other clinicopathological risk factors. Our radiomics-based classifier demonstrated preferable sensitivity and AUC compared with the corresponding values in previous studies (29, 35). Thus, the radiomics-based classifier can improve prognostic performance when combined with clinicopathological prognostic features. Moreover, the strong predictive performance of the Rad-score may be attributable to finding that, unlike previously reported clinicopathological covariates and molecular biomarkers, the radiomics signature may be an effective approach by which intratumor heterogeneity can be quantified and visualized (36, 37).

On this basis, we further presented a recurrence prediction nomogram, which achieved favorable prediction capacity with an AUC of 0.954 in the training cohort and 0.906 in the validation cohort by integrating both the radiomics signature and clinicopathological variables. According to the nomogram-predicted probability, for CRC patients with a high risk of disease recurrence, closer follow-up and postoperative adjuvant chemotherapy are required to achieve an enduring benefit from surgery. Patients with a low risk may not only avoid unnecessary medical examinations and therapy but may also experience a reduced burden of follow-up costs. Therefore, both clinicians and patients could benifit from this scoring system, which may be an effective tool for personalized prediction of the risk of disease recurrence (38).

Notably, our results indicated that chemotherapy is not an independent predictor of risk in the training and validation cohorts. Previous study result the similar conclusion, which demonstrated that adjuvant chemotherapy was ineffective for patients with stage II CRC, regardless of the presence of any poor prognostic factors (12). Fu et al. (39) also indicated that chemotherapy did not improve survival in all patients and might even be associated with poorer cancer-specific survival outcomes. In the present study, we were able to use the radiomics-based classifier to stratify patients into low- and high-risk groups based on significantly different DFS rates. In addition, our radiomics-based classifier successfully identified stage II CRC patients who were most likely to benefit from adjuvant therapy. Further use of this classifier should allow us to more comprehensively assess cancer risk and might be beneficial for therapeutic decision-making.

There exist some limitations of this study. First, as a retrospective study, there was unavoidable bias related to recruitment from a single center and specialized cancer center. As such, standard protocols for imaging parameters, contrast agents, and the methods of texture analysis are generally needed to facilitate further multicenter research. Second, other phases of CT images were not discussed in the current study. These images may better represent more potential tumor heterogeneity and require further investigation. Last, we semiautomatically segmented all lesions, which was time-consuming. Therefore, deep learning method have potential for automatic segmentation of lesions in further research.

In summary, our pilot study presented and validated a combined model that integrated radiomics signature and clinicopathological risk factors for risk classification in stage II CRC. Radiomic features may provide complementary prognostic value to the traditional clinicopathological risk factors and allow for the better stratification of patients receiving adjuvant therapy, thereby helping clinicians assess patient prognosis and guide personalized treatment. However, use of this model will require further external validation before its widespread implementation in clinical practice.

## Data Availability Statement

The original contributions presented in the study are included in the article/Supplementary Material, further inquiries can be directed to the corresponding author/s.

## Ethics Statement

The studies involving human participants were reviewed and approved by Tianjin Medical University Cancer Institute and Hospital. Written informed consent for participation was not required for this study in accordance with the national legislation and the institutional requirements.

## Author Contributions

LZ, WM, and SF: study design and methods development. JQ, QS, XC, and SF: lesions identification and marking. SF, WM, and CL: results inference and implementation of methods. SF, WM, and XL: manuscript writing. SF, XC, CL, XL, LZ, QS, JQ, WM, and ZY: manuscript/results proof read and approval. All authors contributed to the article and approved the submitted version.

## Conflict of Interest

The authors declare that the research was conducted in the absence of any commercial or financial relationships that could be construed as a potential conflict of interest.

## Abbreviations

CRC: colorectal cancer
AUC: area under the curve
DFS: disease-free survival
LVI: lymphovascular invasion
PNI: perineural invasion
TILs: tumor-infiltrating lymphocytes
NCCN: National Comprehensive Cancer Network
IOP: internal obstruction or perforation
CEA: carcinoembryonic antigen
CA242: carbohydrate antigen 242
CA724: carbohydrate antigen 724
CA199: carbohydrate antigen 199
WHO: World Health Organization
MMR: mismatch repair
MSI: microsatellite instability
PACS: picture archiving and communication system
VOIs: volume of interest
RFs: radiomics features
ROI: region of interest
GLCM: gray level co-occurrence matrix-based features
GLRLM: gray level run-length matrix-based features
GLSZM: gray level size zone matrix
NGTDM: neighboring gray tone difference matrix
GLDM: gray level dependence matrix
ICCs: interclass correlation coefficients
LASSO: least absolute shrinkage and selection operator
ROC: receiver operating characteristic
SMOTE: synthetic minority over-sampling technique.
