# Supplementary material for: CT-Based Radiomics Signature: A Potential Biomarker for Predicting Postoperative Recurrence Risk in Stage II Colorectal Cancer
Source: Front Oncol. 2021 Mar 19;11:644933. doi: 10.3389/fonc.2021.644933 (PMC8017337; doi:10.3389/fonc.2021.644933)
Supplement: Supplementary file 1 [file Table_1.DOCX]

**Table S1. Radiomics features selected in Lasso regression analysis**

| **Intercept and radiomics features** | **Coefficients** |
| --- | --- |
| Intercept | -1.265858e+01 |
| original_shape_Maximum2DDiameterColumn | 6.764585e+00 |
| original_shape_Maximum2DDiameterSlice | -1.519925e+00 |
| original_firstorder_Median | -2.306216e+01 |
| original_glcm_InverseVariance | -1.746119e+01 |
| original_glcm_MaximumProbability | 1.975995e+01 |
| original_glrlm_LongRunHighGrayLevelEmphasis | 7.942853e+00 |
| original_glrlm_RunEntropy | -2.435239e+01 |
| original_gldm_SmallDependenceLowGrayLevelEmphasis | -1.610079e+01 |
| log.sigma.1.0.mm.3D_firstorder_90Percentile | -2.914244e+00 |
| log.sigma.1.0.mm.3D_firstorder_Maximum | -5.516299e+00 |
| log.sigma.1.0.mm.3D_firstorder_Skewness | 8.359889e+00 |
| log.sigma.1.0.mm.3D_glcm_ClusterShade | 2.297919e+01 |
| log.sigma.1.0.mm.3D_glcm_Idmn | -9.497864e+00 |
| log.sigma.1.0.mm.3D_glcm_MaximumProbability | -1.148761e+01 |
| log.sigma.1.0.mm.3D_glszm_LargeAreaLowGrayLevelEmphasis | -1.027197e+01 |
| log.sigma.1.0.mm.3D_glszm_SmallAreaEmphasis | -1.342599e+01 |
| log.sigma.1.0.mm.3D_gldm_DependenceEntropy | -8.706796e+00 |
| log.sigma.2.0.mm.3D_firstorder_Skewness | -6.110064e-01 |
| log.sigma.2.0.mm.3D_glcm_Correlation | 1.127200e+01 |
| log.sigma.2.0.mm.3D_glcm_Idmn | -5.554283e+00 |
| log.sigma.2.0.mm.3D_glcm_InverseVariance | -4.702028e+00 |
| log.sigma.2.0.mm.3D_glszm_GrayLevelNonUniformity | -1.052165e+01 |
| log.sigma.2.0.mm.3D_gldm_DependenceVariance | 9.383952e+00 |
| log.sigma.2.0.mm.3D_gldm_SmallDependenceLowGrayLevelEmphasis | -1.862927e+01 |
| log.sigma.2.0.mm.3D_ngtdm_Contrast | 1.723113e+01 |
| log.sigma.3.0.mm.3D_firstorder_90Percentile | 1.370428e+01 |
| log.sigma.3.0.mm.3D_glcm_ClusterShade | 9.944904e+00 |
| log.sigma.3.0.mm.3D_glcm_Correlation | 4.104602e+00 |
| log.sigma.3.0.mm.3D_glcm_InverseVariance | 3.543057e-01 |
| log.sigma.3.0.mm.3D_glszm_SizeZoneNonUniformity | -1.308445e+01 |
| log.sigma.3.0.mm.3D_glszm_SmallAreaEmphasis | 9.652887e-01 |
| log.sigma.3.0.mm.3D_gldm_DependenceVariance | 5.850953e+00 |
| log.sigma.3.0.mm.3D_gldm_LargeDependenceHighGrayLevelEmphasis | -4.509619e+00 |
| wavelet.LLH_firstorder_Maximum | 1.709737e+00 |
| wavelet.LLH_firstorder_Skewness | 2.214537e+00 |
| wavelet.LLH_glcm_ClusterShade | -7.649177e+00 |
| wavelet.LLH_glcm_Correlation | -1.165118e+01 |
| wavelet.LLH_glcm_Idmn | -9.745379e+00 |
| wavelet.LLH_glszm_SmallAreaEmphasis | 1.352165e+01 |
| wavelet.LHL_firstorder_Skewness | 1.236594e+01 |
| wavelet.LHL_glcm_ClusterProminence | -1.918126e+01 |
| wavelet.LHL_glcm_ClusterShade | -2.143186e+01 |
| wavelet.LHL_glcm_Correlation | -6.284263e+00 |
| wavelet.LHL_gldm_DependenceVariance | -3.703200e+00 |
| wavelet.LHL_ngtdm_Contrast | 2.833058e+01 |
| wavelet.LHH_firstorder_Skewness | 6.701715e+00 |
| wavelet.LHH_glcm_ClusterShade | -1.746246e+01 |
| wavelet.LHH_glcm_Correlation | 5.682917e+00 |
| wavelet.LHH_glcm_Imc1 | 2.298131e+01 |
| wavelet.LHH_glrlm_LongRunHighGrayLevelEmphasis | -6.979382e-01 |
| wavelet.LHH_glszm_SmallAreaEmphasis | 1.024298e+01 |
| wavelet.LHH_glszm_ZoneEntropy | -7.753833e+00 |
| wavelet.LHH_gldm_SmallDependenceLowGrayLevelEmphasis | -4.107987e+00 |
| wavelet.HLL_firstorder_Maximum | -1.567858e+00 |
| wavelet.HLL_firstorder_Skewness | -3.502314e+00 |
| wavelet.HLL_glcm_Correlation | -1.805877e+00 |
| wavelet.HLL_glszm_SmallAreaLowGrayLevelEmphasis | 8.253632e-02 |
| wavelet.HLH_firstorder_Kurtosis | 8.044303e+00 |
| wavelet.HLH_firstorder_Median | 8.790537e-01 |
| wavelet.HLH_glcm_ClusterProminence | 2.239153e+01 |
| wavelet.HLH_glcm_Imc1 | -2.017841e-05 |
| wavelet.HLH_glcm_Idn | 6.035003e+00 |
| wavelet.HLH_gldm_LargeDependenceHighGrayLevelEmphasis | 6.661834e-01 |
| wavelet.HHL_firstorder_Mean | 4.404267e+00 |
| wavelet.HHL_firstorder_Median | 1.455255e+00 |
| wavelet.HHL_glcm_JointAverage | -2.566700e+00 |
| wavelet.HHL_glcm_Correlation | -8.052429e+00 |
| wavelet.HHL_glszm_LargeAreaLowGrayLevelEmphasis | 8.514156e-02 |
| wavelet.HHL_glszm_SmallAreaEmphasis | 9.104316e-01 |
| wavelet.HHL_glszm_SmallAreaLowGrayLevelEmphasis | 6.277902e+00 |
| wavelet.HHL_gldm_DependenceVariance | 7.504797e+00 |
| wavelet.HHH_firstorder_Mean | 2.804131e+00 |
| wavelet.HHH_glcm_Correlation | 7.959606e+00 |
| wavelet.HHH_glszm_LargeAreaLowGrayLevelEmphasis | 1.526295e+01 |
| wavelet.HHH_glszm_SizeZoneNonUniformityNormalized | -9.684248e+00 |
| wavelet.HHH_glszm_SmallAreaLowGrayLevelEmphasis | -1.246789e+01 |
| wavelet.HHH_gldm_DependenceNonUniformityNormalized | -1.181030e+01 |
| wavelet.HHH_gldm_SmallDependenceLowGrayLevelEmphasis | -4.662333e+00 |
| wavelet.LLL_firstorder_InterquartileRange | -1.158843e+01 |
| wavelet.LLL_glcm_MaximumProbability | 4.213483e+00 |
| wavelet.LLL_glrlm_RunEntropy | 6.190597e+01 |
| wavelet.LLL_glszm_LargeAreaLowGrayLevelEmphasis | 4.417236e+00 |
| wavelet.LLL_glszm_SmallAreaLowGrayLevelEmphasis | 3.737390e+00 |
| wavelet.LLL_glszm_ZoneEntropy | -1.025563e+01 |
| wavelet.LLL_gldm_DependenceVariance | -4.224636e+01 |
| gradient_firstorder_Minimum | -5.612561e+00 |
| gradient_glcm_Idn | 2.384797e+01 |
| gradient_glrlm_RunLengthNonUniformity | 1.183681e+01 |
| gradient_gldm_DependenceNonUniformityNormalized | 5.257757e+00 |
| gradient_ngtdm_Coarseness | -3.889516e+00 |
| gradient_ngtdm_Strength | -8.412691e+00 |
| square_firstorder_InterquartileRange | 2.551351e+00 |
| square_firstorder_Minimum | 8.573311e-01 |
| square_glcm_ClusterProminence | -2.678230e+01 |
| square_glcm_Imc1 | -4.756509e+00 |
| square_glcm_Imc2 | 2.715970e+01 |
| square_glcm_Idmn | -2.259509e+01 |
| square_glcm_InverseVariance | -9.237315e+01 |
| square_glszm_ZoneVariance | 1.076466e+01 |
| square_ngtdm_Busyness | -6.875486e+00 |
| square_ngtdm_Contrast | 4.508213e+01 |
| squareroot_firstorder_Kurtosis | 1.387341e+01 |
| squareroot_firstorder_Median | 2.032050e+01 |
| squareroot_glcm_ClusterShade | 6.411092e+00 |
| squareroot_glcm_Idn | -1.813028e+01 |
| squareroot_glszm_SmallAreaLowGrayLevelEmphasis | -5.109655e+00 |
| squareroot_gldm_LargeDependenceHighGrayLevelEmphasis | 5.959638e+00 |
| exponential_firstorder_Skewness | -2.915385e+01 |
| exponential_glszm_ZoneVariance | -1.373583e+01 |
| logarithm_glszm_ZoneEntropy | -1.541798e+01 |
| logarithm_gldm_LargeDependenceHighGrayLevelEmphasis | -7.397559e+00 |
| logarithm_gldm_LargeDependenceLowGrayLevelEmphasis | 1.623574e+01 |
| logarithm_ngtdm_Strength | 3.303488e+00 |
