# Supplementary material for: CT-Based Radiomics Signature: A Potential Biomarker for Predicting Postoperative Recurrence Risk in Stage II Colorectal Cancer
Source: Front Oncol. 2021 Mar 19;11:644933. doi: 10.3389/fonc.2021.644933 (PMC8017337; doi:10.3389/fonc.2021.644933)
Supplement: Supplementary file 2 [file Table_2.DOCX]

**Table S2. Univariate and multivariate cox regression analysis of disease-free survival in the validation cohort**

| **Variable** | **Univariate analysis** | | | **Multivariate analysis** | | |
| --- | --- | --- | --- | --- | --- | --- |
|  | **HR** | **95%CI** | ***P* Value** | **HR** | **95%CI** | ***P* Value** |
| Gender | 0.679 | 0.258-1.789 | 0.434 |  |  |  |
| Age | 1.069 | 1.017-1.122 | 0.0082^*^ | 1.092 | 1.012-1.179 | 0.024^*^ |
| Histologic grade | 1.432 | 0.690-2.974 | 0.335 |  |  |  |
| Location | 0.977 | 0.717-1.331 | 0.882 |  |  |  |
| Smoking | 2.648 | 1.041-6.737 | 0.041^*^ | 0.677 | 0.118-3.879 | 0.662 |
| Hypertension | 0.175 | 0.023-1.314 | 0.090 |  |  |  |
| Family history of cancer | 2.588 | 0.983-6.816 | 0.054 |  |  |  |
| Diabetes | 0.635 | 0.147-2.749 | 0.544 |  |  |  |
| CEA level | 2.776 | 1.091-7.061 | 0.032^*^ | 1.576 | 0.530-4.685 | 0.413 |
| CA242 | 2.302 | 0.906-5.850 | 0.080 |  |  |  |
| CA724 | 1.630 | 0.587-4.527 | 0.349 |  |  |  |
| CA199 | 2.149 | 0.816-5.660 | 0.121 |  |  |  |
| Ki-67 level | 1.005 | 0.975-1.037 | 0.743 |  |  |  |
| T stage | 1.658 | 0.673-4.081 | 0.272 |  |  |  |
| Lymphovascular invasion | 2.618 | 1.030-6.655 | 0.043^*^ | 8.007 | 1.126-56.966 | 0.038^*^ |
| Perineural invasion | 5.678 | 1.874-17.210 | 0.002^*^ | 1.278 | 0.320-5.111 | 0.729 |
| IOP status | 3.821 | 1.374-10.630 | 0.010^*^ | 1.698 | 0.488-5.909 | 0.406 |
| Number of nodes examined | 3.384 | 1.280-8.942 | 0.014^*^ | 2.153 | 0.633-7.321 | 0.220 |
| Mismatch repair status | 0.334 | 0.077-1.448 | 0.143 |  |  |  |
| Adjuvant chemotherapy | 4.631 | 1.070-20.050 | 0.040^*^ | 2.560 | 0.429-15.289 | 0.303 |
| Rad-score | 24.41 | 5.928-100.50 | <0.001^*^ | 60.715 | 6.616-557.147 | <0.001^*^ |

Note: CEA, carcinoembryonic antigen; CA242, carbohydrate antigen 242; CA724, carbohydrate antigen 724; CA199, carbohydrate antigen199; IOP status, internal obstruction or perforation.

*, *P* <0.05
