# Supplementary material for: CT-Based Radiomics Signature: A Potential Biomarker for Predicting Postoperative Recurrence Risk in Stage II Colorectal Cancer
Source: Front Oncol. 2021 Mar 19;11:644933. doi: 10.3389/fonc.2021.644933 (PMC8017337; doi:10.3389/fonc.2021.644933)
Supplement: Supplementary file 3 [file Data_Sheet_1.DOCX]

**Supplemental Material**

1. **CT scanning protocol**
2. CT examinations were conducted using a Somatom Sensation 64 (Siemens Medical Solutions, Forchheim, Germany) or Discovery CT750 HD scanner (GE Medical Systems, Milwaukee, WI).
3. The mean acquisition parameters of the images were tube voltage 120 kVp, tube current 150-200 mA, slice thickness 1.25 mm, slice interval 1.25 mm, field of view 35–50 cm, matrix 512 × 512, rotation time 0.7 s, and pitch 0.969.
4. Following an unenhanced scan, 1.2 mL/kg nonionic contrast media iohexol (Omnipaque 300, GE Healthcare, USA) was injected with a power injector at a rate of 2.5 mL/s through the median cubital vein. This was followed by 20 mL saline flushing at a rate of 3.0 mL/s. Then, images were obtained with a postinjection delay of 30 s and 70 s after initiation of contrast material injection, corresponding to the arterial and venous phases, respectively.
5. **CT image feature extraction**

A total of 1561 radiomic features were drawn from each segmented lesion and can grouped as follows: (1) Shape-based features (14 features): these include descriptors of three-dimensional size and shape of the region of interest (ROI), which are dependent from the gray level intensity distribution in the ROI and therefore only calculated on the non-derived image and mask; (2) First-order statistics features (306 features): these are calculated from gray-level histogram and describe the distribution of voxel intensities within the image region; (3) gray level co-occurrence matrix-based features (GLCM) (374 features) [1]; (4) gray level run-length matrix-based features (GLRLM) (272 features) [2]; (5) gray level size zone matrix (GLSZM) (272 features) [3], (6) neighboring gray tone difference matrix (NGTDM) (85 features) [3] (7) gray level dependence matrix (GLDM) (238 features) .

The total number of radiomic features could be calculated as:

$1$4+306+374+272+272+85+238=1561

1. **Synthetic Minority Over-sampling Technique (SMOTE)**

When data are imbalance for case and controls, classification can be biased in favor of the majority class. To overcome this problem, we applied SMOTE[4] to increase the number of samples in our minority class ( recurrence group) by creating “synthetic” examples. Five nearest neighbors were used as SMOTE parameter and minority classes were subsampled to be equal numbered with the majority samples.

1. **Radiomics score**

The radiomics signature was constructed based on the regression analysis with a radiomics score calculated for each patient. The formula to calculate the score of radiomics signature is

*Score* = *Intercept* + *Coefficient* × *Radiomics features*.

**REFERENCES**

1. Haralick RM, Shanmugam K, Dinstein I (1973) Textural Features for Image Classifcation. IEEE Transactions on Systems, Man, and Cybernetics 1973; 3:610–621.
2. Galloway MM. Texture analysis using grey level run lengths. NASA STI/Recon Technical Report N.1974 Jul;75.
3. Srinivasan GN, Shobha G. Statistical texture analysis. Proceedings of World Academy of Science Engineering ＆ Technolog, 2008, 36: 1264-1269．
4. Chawla NV, Bowyer KW, Hall LO, et al. SMOTE: Synthetic minority over-sampling technique. J Artif Intell *Res* 2002;16:321-357
