# Supplementary figures and images for: CT-Based Radiomics Signature: A Potential Biomarker for Predicting Postoperative Recurrence Risk in Stage II Colorectal Cancer
Source: Front Oncol. 2021 Mar 19;11:644933. doi: 10.3389/fonc.2021.644933 (PMC8017337; doi:10.3389/fonc.2021.644933)

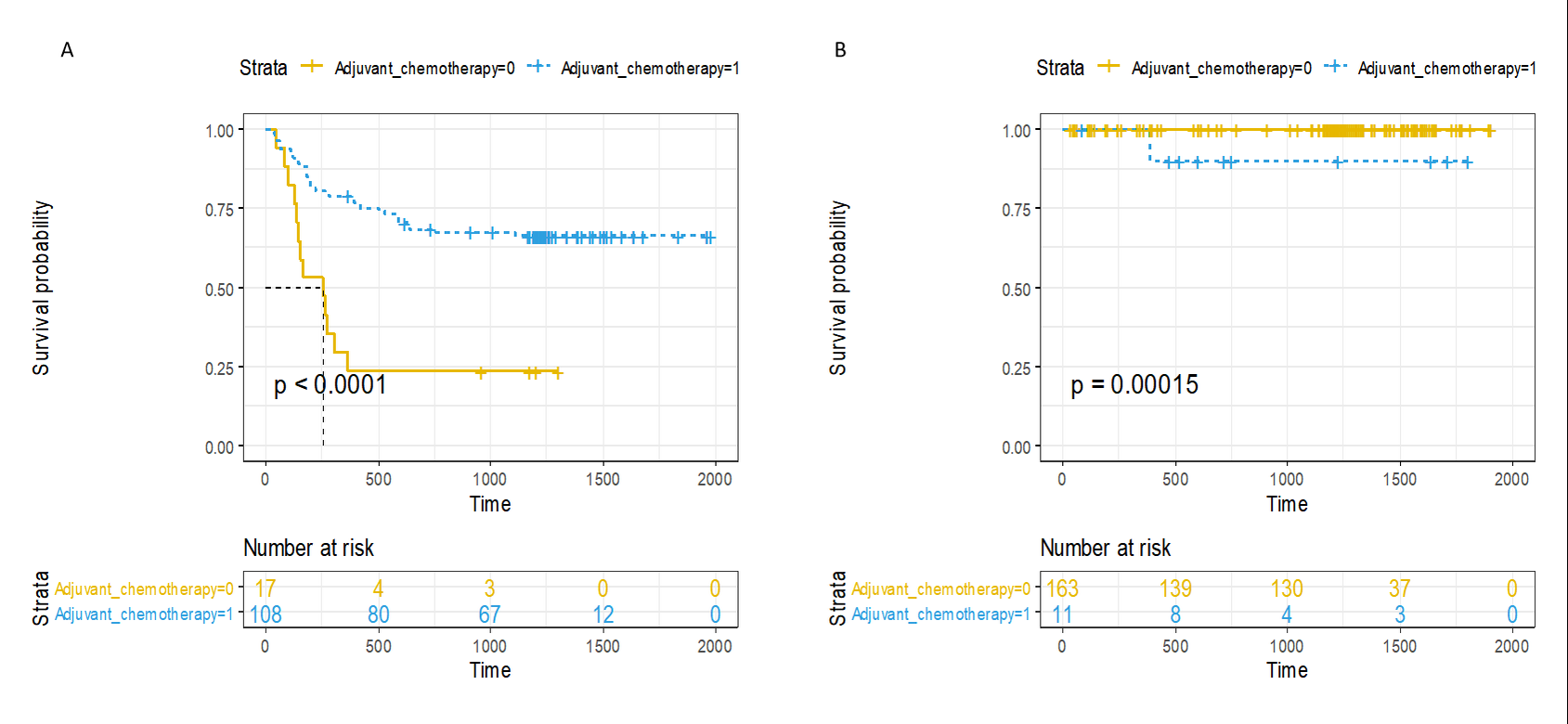

Supplement: Supplementary file 4 [file Image_1.TIF]
