# Supplementary material for: CT-Based Radiomics Signature: A Potential Biomarker for Predicting Postoperative Recurrence Risk in Stage II Colorectal Cancer
Source: Front Oncol. 2021 Mar 19;11:644933. doi: 10.3389/fonc.2021.644933 (PMC8017337; doi:10.3389/fonc.2021.644933)
Supplement: Supplementary file 5 [file Data_Sheet_2.DOCX]

**Figure S1:** Kaplan-Meier curves for patients in different subgroups, which were stratified by the receipt of chemotherapy. (A) Radiomics-based classifier defined high risk group. (B) Radiomics-based classifier defined low risk group.
